# Identity of tendon stem cells – how much do we know?

**DOI:** 10.1111/jcmm.12007

**Published:** 2012-12-28

**Authors:** Pauline Po Yee Lui

**Affiliations:** a Department of Orthopaedics and Traumatology, Faculty of Medicine, The Chinese University of Hong KongHong Kong, SAR, China; b The Hong Kong Jockey Club Sports Medicine and Health Sciences Centre, Faculty of Medicine, The Chinese University of Hong KongHong Kong, SAR, China; c Program of Stem Cell and Regeneration, School of Biomedical Science, The Chinese University of Hong KongHong Kong, SAR, China

**Keywords:** tendon stem cells, tendon injury, *in vivo* identity, stem cell niche

## Abstract

Tendon stem cells are multi-potent adult stem cells with broad differentiation plasticity that render them of great importance in cell-based therapies for the repair of tendons. We called them tendon-derived stem cells (TDSCs) to indicate the tissue origin from which the stem cells were isolated *in vitro*. Based on the work of other sources of MSCs and specific work on TDSCs, some properties of TDSCs have been characterized / implicated *in vitro*. Despite these findings, tendon stem cells remained controversial cells. This was because MSCs residing in different organs, although very similar, were not identical cells. There is evidence of differences in stem cell-related properties and functions related to tissue origins. Similar to other stem cells, tendon stem cells were identified and characterized *in vitro*. Their *in vivo* identities, niche (both anatomical locations and regulators) and roles in tendons were less understood. This review aims to summarize the current evidence of the possible anatomical locations and niche signals regulating the functions of tendon stem cells *in vivo*. The possible roles of tendon stem cells in tendon healing and non-healing are presented. Finally, the potential strategies for understanding the *in vivo* identity of tendon stem cells are discussed.

- Introduction
- Stem cell niche and its clinical implications
- Where do tendon stem cells populate?
  – Vasculature as a perivascular niche for MSC
  – Vascular and non-vascular sources of tendon stem cells
- Possible niche signals regulating tendon stem cells
  – Oxygen tension
  – Extracellular matrix
  – Mechanical loading
  – Biological factors
  – Interstitial cells
- Possible sources and roles of tendon stem cells in tendon healing and failed healing
  – Evidence that tendon stem cells participate in tendon healing or failed healing
  – Sources of TDSCs that contribute to tendon healing or failed healing
  – Potential functions of tendon stem cells
- Possible strategies to track the in vivo identity of tendon stem cells
- Conclusion

## Introduction

Mesenchymal stem cells (MSCs) are multi-potent cells that have the capacity to develop into different mature mesenchymal cell types. Recently, we and others have isolated stem cells from tendon tissues of various species *in vitro* [1–4]. These cells expressed stem cell-related markers, formed adherent colonies in culture and showed self-renewal potential [1–3]. They could differentiate into osteogenic, chondrogenic and adipogenic lineages upon induction *in vitro* and could form tendon-like, cartilage-like, bone-like and tendon–bone junction-like tissues after subcutaneous transplantation in nude mouse or nude rat models [1, 2]. We called these cells tendon-derived stem cells (TDSCs) to indicate the tissue origin from which the stem cells were isolated *in vitro*. Although MSCs isolated from different tissues share some common stem cell properties, they might exhibit some tissue-specific characteristics and hence functional differences [5]. Our recent data showed that TDSCs exhibited higher clonogenicity, proliferation, multi-lineage differentiation potential compared with paired bone marrow-derived MSCs (BMSCs) *in vitro* [6]. Compared with BMSCs, TDSCs also expressed higher levels of BMP receptor IA, IB and II, as well as showed higher BMP-2-induced osteogenic differentiation [7]. This data supported that TDSCs and BMSCs might be distinct cell types. However, the possibility that TDSCs and BMSCs were derived from a common ancestor that gradually assumed tissue-specific phenotypes under the influence of local niche could not be excluded. While many stem cell-related markers were reported to be expressed by TDSCs, none of them were specific, which could uniquely identify TDSCs *in vitro* and tendon stem cells *in vivo*. Moreover, the markers expressed by tendon stem cells *in vivo* might be altered as a consequence of *in vitro* cell culture. Therefore, tendon stem cells *in vivo* remained controversial cells. Compared with the *in vitro* characteristics, their *in vivo* identities, niche and roles in tendon were less understood. *C*ultured tendon stem cells have been reported to promote early tendon healing in animal models [8, 9]. However, the conditions were not optimized. To facilitate the design of new drugs for modulating the functions of endogenous tendon stem cells or optimize the conditions for *ex vivo* culture of tendon stem cells for drug testing and tendon repair, better understanding of the *in vivo* identities, niches and roles of stem cells in tendon is essential. In this review, I have summarized the possible anatomical locations and niche signals regulating the functions of tendon stem cells *in vivo*. The possible roles of tendon stem cells in tendon healing and non-healing are presented. Finally, the potential strategies for understanding the *in vivo* identity of tendon stem cells are discussed. As stem cells isolated *in vitro* might exhibit differences compared with stem cells *in vivo*, and different names have been used to refer to stem cells isolated from tendon tissue *in vitro*, I have used the term ‘tendon stem cells’ to refer to stem cells and progenitor cells in tendon tissue *in vivo* and ‘TDSCs’ to refer to stem and progenitor cells isolated from tendon tissue *in vitro*, respectively, in this review.

## Stem cell niche and its clinical implications

Mesenchymal stem cells have limited function without the niche. Stem cell niches are more than simple histological and anatomical locations. They are dynamic well-orchestrated 3D microenvironments that interact and regulate fates (quiescence, self-renewal or differentiation) of adult stem cells through the action of cellular (physical contact) and non-cellular (regulatory factors) components. The removal of MSCs from their native environment during *in vitro* cell culture might explain the reduced multi-lineage differentiation potential of adult stem cells, such as TDSCs, during subculture [10]. As MSCs, including tendon stem cells, are defined by their ability to self-renew and generate different cells of mesodermal lineage, identification of anatomical and functional niche components that maintain their ‘stemness’ properties and regulate their differentiation is important. This is not only for understanding the MSC biology *in vivo* but also for the practical purpose of mobilizing endogenous MSCs and producing sufficient quantities of MSCs replicating *in vivo* characteristics *in vitro* for therapeutic applications. Using TDSC as an *in vitro* model, Zhang *et al*. [11] reported that platelet-rich plasma clot releasate (PRCR)-enhanced TDSC proliferation and differentiation into tenocytes and total collagen production, suggesting that PRCR, which is commonly used clinically for the treatment of tendon injuries and disorders, was likely to be safe and might have promoting effects on tendon healing. Dexamethasone, which is used for the treatment of tendon injuries but is often associated with tendon rupture and impaired tendon healing, reduced the mean colony size and number of low-density tenocyte culture as compared with control cultures, suggesting that dexamethasone might inhibit tendon progenitor cell recruitment [12]. In another study, treatment of human TDSCs with dexamethasone decreased cell proliferation and promoted non-tenocyte differentiation *in vitro*. Dexamethasone treatment therefore might deplete the stem cell pool and lead to the formation of non-tendinous tissues which might make tendon susceptible to rupture [13]. Using an *in vitro* human TDSC culture, Haasters *et al*. [14] reported that bupivacaine, ropivacaine, but not morphine, had a significant cytotoxic effect on human TDSCs, suggesting that morphine might be a better analgesic drug after anterior cruciate ligament reconstruction in clinical practice. Chondrocyte-like cells were observed in clinical samples of tendinopathy [15]. Using tendon explant culture containing stem and progenitor cells isolated from healthy and tendinopathic human tissues, de Mos *et al*. [16] reported that triamcinolone and platelet-rich plasma influenced the chondrogenic gene expression pattern, suggesting that the model can be used to evaluate existing and future treatment opportunities. Through the incorporation of *in vivo* niche factors into the *in vitro* culture system, the *in vitro* TDSC drug-testing system as used in these previous studies could be further improved and more closely reflect the *in vivo* situation.

However, identifying the niche of tendon stem cells and determining how the functions of tendon stem cells are regulated by the local niche is experimentally challenging because of the lack of specific marker(s) for the tracking of tendon stem cells. Below I presented evidence about the possible anatomical location(s) (section ‘Where do tendon stem cells populate?’) and regulatory factors (section ‘Possible niche signals regulating tendon stem cells’) that might regulate the fate of tendon stem cells.

## Where do tendon stem cells populate?

### Vasculature as a perivascular niche for MSC

Mounting evidence suggested that the wall of capillaries, small vessels and large vessels harboured stem / progenitor cells [17–24]. Some studies further suggested that MSCs were derived from pericytes [19, 25]. Pericytes (also called mural cells) are relatively undifferentiated cells that lie on the abluminal side of small blood vessels, and serve as blood-flow regulators in the microvasculature. Pericytes/ perivascular cells from a variety of tissues were reported to exhibit a phenotype that was strikingly similar to that of MSCs including hypo-immunogenicity, clonogenicity, multi-lineage differentiation potential, long-term multi-potency, migration ability, as well as expression of both MSC markers (*e.g*. CD44, CD90, CD73, CD105, CD166, SSEA-4, Stro-1) and pericyte markers (*e.g*. 3G5, NG2, ALP, CD146, PDGF-β, α-SMA) [17–19, 21, 24, 26–31]. Using a genetic lineage tracing technique, Feng *et al*. [32] has recently demonstrated the direct differentiation of genetically marked pericytes to odontoblasts both during incisor growth and repair following damage. However, another recent study questioned the concept that pericytes and MSCs originate from the same cells [33]. Instead, the authors of the study postulated the existence of a different population of cells with tri-lineage differentiation potential that colocalized with pericytes [33]. Besides small blood vessels, the adventitia of foetal and adult arteries was also suggested as a niche for stem/progenitor cells. The adventitial cell forms the outermost layer of large blood vessels and functions as a dynamic compartment for cell trafficking into and out of the artery wall [34]. The data in the recent literature confirmed the existence of multi-potent MSCs within the vascular adventitia [35–38]. For example, Hoshino *et al*. [35] identified progenitor cells in the adventitia of human pulmonary arteries that expressed mesenchymal stem/progenitor cell markers, but were negative for endothelial and hematopoietic cell markers, and showed multi-lineage differentiation potential.

The perivascular localization of MSCs has important biological and clinical implications. First, it might explain the ubiquitous distribution of MSCs throughout the body. Second, the proximity of MSCs to blood vessels suggested that they might be uniquely poised to respond to the regulatory signals from the vascular system related to tissue injury or disorder, both local and distal to the injured site, and this might provide a route for modulating MSC function for the promotion of tissue healing and treatment of tissue disorder.

### Vascular and non-vascular sources of tendon stem cells

For tendons, there was also evidence that the vasculature of tendon tissue might harbour stem cells. Based on *in vivo* and *in vitro* studies, it has been demonstrated that perivascular cells of human supraspinatus tendon capillaries expressed both tendon cell markers (Scx, collagen type I, collagen type III, smad8) and stem/precursor cell markers (CD133, Musashi-1, Nestin, CD44, CD29), in addition to the pericyte-associated marker α-SMA [39]. Using stem cell markers, SSEA-4 and Sca-1, Mienaltowski and Bir 40 reported that tendon stem cells were localized mainly at the paratenon surrounding the rat Achilles tendon, where most blood vessels were present compared with the tendon proper. Our results also showed that more iododeoxyuridine (IdU) label-retaining cells (LRC) were observed in the peritenon compared with the tendon proper, and some, but not all, LRC, were observed at the perivascular regions in the peritenon in rat patellar tendons 41.

However, tendon proper is hypovascular compared with other tissues and receives its blood supply mainly from the endotenon and paratenon [42]. Rat TDSCs, although positive for pericyte marker α-SMA as shown by immunocytochemical staining [3], did not show surface expression of CD146, PGDFR-β and NG-2 during *in vitro* culture as shown by flow cytometry (unpublished results). Cultured human TDSCs also did not express the pericyte marker CD146 on the cell surface as shown by flow cytometry [43]. Bi *et al*. [1] also demonstrated the null surface expression of CD106, a vascular cell marker expressed by BMSCs, in TDSCs *in vitro*. Tendon stem cells either might have lost the pericyte markers during *in vitro* subculture and/or there might be more than one source of tendon stem cells in tendons, and TDSCs used in our study might represent the non-vascular source of tendon stem cells. The TDSCs used in our laboratory were isolated from the tendon proper after removing the peritenon which contained the majority of the blood vessels. Bi *et al*. [1] reported the residence and alignment of LRC in-between long parallel collagen fibrils containing biglycan and fibromodulin and no vasculature was observed in the images provided in the report [1]. Consistent with this finding, we observed the presence of LRC between parallel collagen fibrils in the tendon proper in rat patellar tendons 41. Indeed, besides the perivascular regions, stem/precursor cell-related markers such as nestin and Musashi-1 were also detected in tendon cells embedded in dense extracellular matrix (ECM) [39]. This finding supported that there might be a non-vascular source of stem cells in tendons. Recently, Mienaltowski *et al*. [44] reported the isolation of two different populations of stem/progenitor cells from the peritenon and tendon proper of mouse Achilles tendons. Both cell sources were negative for the perivascular surface marker CD133. Although both stem/progenitor cell populations were multi-potent, only cells isolated from the tendon proper were able to produce a calcified matrix. The stem/progenitor cells isolated from the peritenon showed higher mRNA level of endomucin (a vascular marker), but low level of tenomodulin and scleraxis, relative to the cells isolated from the tendon proper. The authors hence suggested that different stem/progenitor cell populations existed within distinct niches at the tendon proper and peritenon; and the stem/progenitor cells in the peritenon might be more vascular in origin. Kurth *et al*. [45] reported that MSCs identified *in vivo* in the knee joint synovium were distinct from pericytes. These MSCs proliferated and differentiated into chondrocytes in areas of cartilage metaplasia within the synovium following articular cartilage injury [45]. In support of these previous findings, Feng *et al*. [32] also reported a non-pericyte origin in addition to a pericyte origin of MSCs in dental pulp. Only a small percentage of odontoblasts were derived from pericytes during incisor growth and repair following damage [32]. A population of MSC-like cells was observed to directly migrate from the cervical end of incisor towards the damaged area, differentiate and contribute to the majority of odontoblasts [32]. The contribution of perivascular niche to the regulation of MSC fate in any given tissue therefore might be variable and might depend on the extent of vascularity. In tissues with low vascularity, such as tendon, the contribution of perivascular niche to the regulation of MSC fate might be less than in tissues with more extensive blood supplies.

We found that LRC in tendons was a heterogeneous cell population as none of the individual MSC markers tested labelled all the LRC and there were non-LRC that were positive for MSC markers (unpublished results). Functionally distinct subsets of LRC hence might exist in tendons. Using immunofluorescence labelling, CD146^+^ and α-SMA^+^ cells were found throughout tendon tissue, in addition to blood vessels, in rat patellar tendons 41. Most of the LRC, including those that were not localized at the blood vessels, were positive for CD146 and α-SMA but not *vice versa*, suggesting that LRC, whether residing close to blood vessels or not, were pericyte-like 41. Whether the non-perivascular but pericyte-like LRC represent cells that have migrated from the capillary walls to the surrounding tissue needs further research, which will have important implications of mobilizing tendon stem cells distal to the vasculature for the promotion of tendon healing and treatment of tendon disorder *via* the vascular system.

## Possible niche signals regulating tendon stem cells

### Oxygen tension

Hypoxia is a potent suppressor of mitochondrial oxidation [46] and has been shown to promote ‘stemness’ in adult and embryonic stem cells (ESCs) [47–50]. There has been no report on the measurement or mathematical estimation of oxygen tension in human tendons. However, it is reasonable to speculate that the oxygen tension inside tendons is low as it has a poor blood supply, which is only about one third that of muscles [51]. The tendon milieu is therefore expected to be hypoxic. Comparison of human TDSCs cultured in hypoxic *versus* normoxic conditions (2% and 20% oxygen tension) showed that the proliferative capacity of human TDSCs was better maintained (25% higher) in the former condition [43]. In addition, hypoxia doubled the number of colony-forming unit-fibroblasts (CFU-F) present at day 14, while reversibly suppressed the differentiation of TDSCs which was thought to be pivotal in the maintenance of stemness [43]. This data suggested that hypoxia enhanced not only the proliferative capacity but also the plasticity of TDSCs. The culture of TDSCs under a hypoxic environment therefore may shorten the time and better maintain the multi-lineage differentiation potential of these cells for tendon repair and drug testing.

### Extracellular matrix

The alteration of the structure and composition of ECM might perturb the balance of cytokines and growth factors stored within the ECM as well as modulate the cell shape and signalling events of tendon stem cells, ultimately affecting their fate. Bi *et al*. [1] showed that biglycan and fibromodulin were important *in vivo* niche components of tendon stem cells as the depletion of biglycan and fibromodulin in a double knock-out mouse model impaired patellar tendon formation [1]. The TDSCs isolated from the double knock-out animal model formed bone-like in addition to tendon-like tissues, whereas wild-type TDSCs only formed tendon-like tissue, suggesting that biglycan and fibromodulin might regulate the fate of tendon stem cells [1]. This has implications of the role of ECM on the pathogenesis of tendinopathy as change in ECM composition with increased proteoglycan deposition is frequently observed in tendinopathic patients [52]. The importance of tendon ECM in the maintenance of the stemness of tendon stem cells was further supported by a recent study which showed that rabbit TDSCs cultured on decellularized tendon matrix showed higher proliferation and better stemness properties, compared with TDSCs cultured on the plastic culture surface [53]. The bioactivity of the decellularized tendon matrix might be as a result of its matrix components and/or bound growth factors. Further study is required to understand the mechanism of the decellularized tendon matrix in maintaining the stemness of TDSCs. Besides composition, the micro-/nano- architecture of the ECM might also provide topographical cues, which might regulate the fate of stem cells. In this regard, the culture of TDSCs in an aligned nanofibrous scaffold promoted their tenogenic commitment, whereas the culture of TDSCs in a random nanofibrous scaffold enhanced their osteogenic differentiation *in vitro* [54]. The cytoskeletal structure of TDSCs might be responsible for mediating their interaction with the ECM [54]. The use of an appropriate substratum or scaffold for TDSC culture *in vitro* can better maintain the *in vivo* properties or promote their tenogenic differentiation for tendon repair.

### Mechanical loading

As tendon functions to transmit load from muscle to bone, cells inside tendon are constantly subjected to mechanical load. Recent findings demonstrated that TDSCs were sensitive to mechanical load [55–57]. Zhang and Wang [56] reported that low cyclic uniaxial mechanical stretching at 4% (‘clamp-to-clamp’ engineering strain) and 0.5 Hz for 12 hrs promoted tenogenic differentiation of TDSCs seeded in microgrooves oriented along the stretching axis, whereas large stretching at 8% and 0.5 Hz induced non-tenogenic differentiation of some TDSCs *in vitro*. Treadmill running with 1 week of training at 13 m/min. for 15 min./day followed by 3 weeks of exercise at 50 min./day and 5 days per week was reported to double the proliferation rate of TDSCs isolated from patellar and Achilles tendons of mice [57]. We reported that cyclic repetitive tensile loading at 4% and 8%, 0.5 Hz, for 4 hrs promoted cell alignment along the loading direction and production of BMP-2 in TDSCs [55]. Results from these studies implied that we might modify the functions of tendon stem cells *in vivo* and hence tendon *via* exercise. Indeed, overuse has been implicated as one of the risk factors for the development of tendinopathy, whereas eccentric exercise has been reported to reduce pain and improve tendon functions [58]. Controlled exercise is also recommended for the promotion of tendon healing after injury. Further research on the response of TDSCs or tendon stem cells to mechanical loading would enable better understanding of the pathogenesis of tendinopathy and the development of optimal exercise protocols to enhance tendon healing while reduce scar tissue formation and tendon adhesions.

### Biological factors

The tendon stem cell fate might be controlled by biological factors such as BMPs and Wnts. TDSCs isolated from the biglycan and fibromodulin double knock-out animal model displayed higher sensitivity to BMP-2 signalling [1]. There was increased expression of chondro-osteogenic BMPs and Wnt3a in the healing tendon cells, chondrocyte-like cells and ossified deposits in the animal model and clinical samples of tendinopathy [15, 59, 60]. Wnt3a promoted the osteogenic differentiation of TDSCs *in vitro* 60, whereas BMP-2 promoted non-tenocyte differentiation and proteoglycan deposition of TDSCs *in vitro* [55, 61]. Because of the multi-potency of tendon stem cells, a molecular defence mechanism might be in place to prevent the erroneous differentiation of tendon stem cells to non-tenocytes. A previous study showed that an activated form of Smad8 protein inhibited the BMP-2-induced osteogenic differentiation of MSCs while promoting their tenogenic differentiation [62]. MSX2 was also reported to act as a molecular defence mechanism for preventing ossification in ligament fibroblasts [63]. Whether these molecules would function to regulate the fate of tendon stem cells requires further research. Study on how biological factors regulate the fate of tendon stem cells *in vivo* would provide information to prevent chondro-ossification and promote healing in tendons.

### Interstitial cells

The behaviour of tendon stem cells might be critically regulated by interaction with neighbouring cells resident in their local microenvironment, both by direct physical contact as well as by secretion of soluble growth factors and cytokines. Tenocytes are the major cell type in tendons and form a three-dimensional network of cell processes throughout tendons [64]. Tenocytes therefore might modulate the fate of tendon stem cells through direct cell–cell contact or production of soluble mediators. In a TDSC–tenocyte mixed culture system, there was higher collagen production in the mixed culture with Achilles or patellar TDSCs isolated from the treadmill running mice compared with TDSCs isolated from cage control mice, supporting the possible interaction between tendon stem cells and tenocytes *in vivo* [57]. As no cell separation was performed in this mixed culture study, it was not clear if the collagen was produced by TDSCs, differentiated TDSCs or tenocytes, and hence the direction of communication and molecular mechanism were not clear. Coculture studies with or without cell–cell contact, followed by cell isolation, might answer this question. Better understanding of the interaction between tendon stem cells and tenocytes *in vivo* may provide information for maintaining the plasticity of TDSCs during *in vitro* culture and for developing new strategies for the promotion of tendon repair.

Telocytes (TC) is a new cell type that has been identified in the stroma of various tissues and organs including heart, skeletal muscle and skin [65]. They are cells with a small cell body with typically 2–3 very long (up to tens / hundreds of μm) and thin (mostly below 0.5 μm) prolongations called the telopodes [65]. They have been overlooked previously, probably because of their thin and winding prolongations that can only be observed under electron microscopy. They are distinct from interstitial fibroblasts by ultrastructure, phenotype and function [65]. While fibroblast functions mainly to produce extracellular matrix proteins, TC promotes intercellular communication either by direct contact *via* junctional proteins or remotely *via* extracellular vesicles [65]. Through their telopodes, TC was found to integrate different cell types for long-distance signalling that was important for cardiac renewing [66]. Therefore, TC is suggested as a stem cell niche. TC has been observed in close proximity to cardiac progenitors of various stages of differentiation [67]. After an experimental myocardial infarction, TC was seen to contribute in tandem with resident stem cells to an increase in the regeneration rate of the cells in the border zone of the infracted area and the surroundings [68]. TC has not been found in tendon. Further research is needed to see if TC can be identified in tendon and its contribution to the tendon stem cell niche.

## Possible sources and roles of tendon stem cells in tendon healing and failed healing

### Evidence that tendon stem cells participate in tendon healing or failed healing

As stem cells reside in tendon tissue, it is logical to expect that tendon stem cells play roles in tendon healing and failed healing after injury. There has been no study that directly addresses the fate and roles of tendon stem cells after tendon injury *in vivo*. TDSCs isolated from a collagenase-induced failed healing patellar tendon injury rat model showed reduced tenogenic capacity compared with TDSCs isolated from healthy tendon [69]. Subsequent analysis further showed that TDSCs isolated from this failed healing model expressed higher levels of BMP-2, BMP-4, BMP-7, BMP receptors, and were more sensitive to BMP-2-induced smad activation compared with TDSCs isolated from healthy tendon, which might contribute to chondro-ossification and failed healing in this animal model 70. This data suggested that TDSCs were likely to participate in tendon healing or failed healing. Further *in vivo* evidence is required to confirm the *in vitro* findings.

### Sources of TDSCs that contribute to tendon healing or failed healing

As the stem cell niche is dynamic, the fate of stem cells and sources contributing to TDSCs isolated from tendons *in vitro* might change, depending on the physiological or pathological status of the tissue. Depending on the stages after tendon injury, stem cells from different sources might be recruited into damaged tendon. Hence the composition of TDSCs *in vitro* might vary, depending on the stages of tendon injury *in vivo*. Mesenchymal cells from nearby tissues and systematic circulation might contribute to tendon healing [71]. A previous study showed that circulation-derived mesenchymal cells and tendon-derived mesenchymal cells contributed to different phases of tendon healing after injury [72]. Using two green fluorescent protein (GFP) chimeric rat models, the authors demonstrated that circulation-derived mesenchymal cells appeared at the initial stage of tendon injury and they were replaced by tendon-derived mesenchymal cells with time [72]. As tendon is connected to muscle, stem and progenitor cells of skeletal muscle [73] might also be a possible source of MSCs for tendon repair after injury. Adipose tissue is an abundant source of MSCs. The infrapatellar fat pad which is located underneath the patellar tendon, therefore, might also be a possible source of MSCs for tendon repair when the patellar tendon is damaged [74]. Compared with TDSCs isolated from healthy tendon, the expression of positive and negative stem cell markers in TDSCs isolated from the collagenase-induced failed healing patellar tendon injury model remained unchanged, except there was lower surface expression of CD73 (60.6% *versus* 98%) and CD44 (63.1% *versus* 79.5%) [69]. The exact origins of isolated TDSCs and tendon stem cells *in vivo* during tendon injury therefore remain obscure.

### Potential functions of tendon stem cells

As MSCs possess multi-lineage differentiation potential, it is generally assumed that they promote tissue repair by direct differentiation into specific cell types. Recent studies showed that MSCs might also promote healing *via* the secretion of immunomodulatory and trophic factors. A number of bioactive molecules secreted by MSCs were capable of promoting cytoprotection, neovascularization, migration, immunoregulation, cell proliferation, ECM synthesis and remodelling [23, 75, 76]. Whether tendon stem cells function to replace the damaged tendon or to establish a regenerative microenvironment for tendon repair is not clear. Both might occur *in vivo* after tendon injury. Our previous study showed that most of the transplanted allogeneic TDSCs were removed from the patellar tendon window wound at week four while promoting tendon repair in a rat model [7]. Very few, if any, TDSCs were present at the wound site and they were positive for proliferating cell nuclear antigen (PCNA) at week four in this animal model (unpublished results).

## Possible strategies to track the *in vivo* identity of tendon stem cells

As information on the *in vivo* niche of tendon stem cells would benefit the mobilization and *ex vivo* culture of tendon stem cells for tendon repair, I will summarize the possible strategies that might be useful for tracking the fate of tendon stem cells *in vivo*. *In vitro* MSC markers could be used to locate positive cells *in vivo* using immunohistochemistry or *in situ* hybridization. This approach, although sensitive, is currently limited by the lack of specific MSC or tendon stem cell markers. Some of the MSC markers such as CD44, CD90, CD73, CD29 and CD105 were not specific to MSCs and were also expressed by fibroblasts [77, 78]. Another approach is to inject labelled cultured stem cells into the circulation to analyse their tissue distribution *in vivo*. This strategy might be less accurate to study the natural distribution of tendon stem cell *in vivo* because the cells might engraft non-specifically in different organs and specific homing signals might be required for recruiting the injected stem cells to tendons. Tendon has a poor blood supply and this might also affect the recruitment of injected cells to healthy tendon. The injection of cultured stem cells directly into an injured tendon is possible, but may not be feasible in an intact tendon with tightly packed and organized collagen fibres. How injection-induced tendon injury may affect the results remains unclear. Dudhia *et al*. 79 compared the amount of labelled BMSCs in tendon lesions of horses with tendinopathies or desmopathies using intralesional, intravenous and regional perfusion routes. They showed that intralesional administration of BMSCs retained the highest number of cells, followed by regional perfusion. Intravenous injection of BMSCs resulted in distribution of cells largely to the lung fields and there were no detectable cells in the tendon lesions 79. Exogenous injection of tendon cells hence might be less accurate in studying the natural distribution and functions of tendon stem cells and it might not reflect or might even disturb the endogenous tendon stem cell activities. The use of bromodeoxyuridine (BrdU) labelling to identify label-retaining cells (LRC) with long cell-cycle time or asymmetric-cell division with non-random chromosomal cosegregation theoretically is useful for the localization of tendon stem cells *in vivo* as they are supposed to be quiescent and retain the label while the differentiated cells proliferate and lose the BrdU signal rapidly during the washout period [80]. Using a double nucleoside analogue cell-labelling system (IdU/CldU), Kurth *et al*. [45] reported the identification of a population of quiescent, slow-cycling, non-hematopoietic, non-endothelial, MSC-like stromal cells, present in both the lining layer and sublining tissue of synovium of knee joint *in vivo*. However, this method is not specific for stem cells and might label cells that have stopped proliferating because of various reasons (*e.g*. differentiation) and hence might be subjected to false-positive errors. The self-renewal capacity of stem cells was suggested to correlate with telomerase activity [81]. Based on this hallmark of stem cells, Breault *et al*. [82] have generated *mTert-*GFP-transgenic mice as a model system to mark male germ cells, hematopoietic stem cells (HSCs) and intestinal crypt cells (ISCs) *in vivo*. The feasibility of using this system to mark stem cells in tendon needs further research. Similar to the BrdU labelling method, this method also does not specifically label resident stem cells in tendons and hence the relative contribution of stem cells from different sources to tendon healing or failed healing cannot be revealed and requires the combined use of tissue-specific markers to elucidate the mechanism. To look systematically for niche in tendon tissue, the ideal method is to mark the tendon stem cells using genetic-based lineage tracing technique and follow their lineages. Information related to tissue development is usually taken into consideration in the selection of appropriate markers for lineage tracing and hence this method is more specific. Using the same approach, Feng *et al*. [32] have used NG2-driven Cre to trace pericytes. Besides, Lounev *et al*. [83] have used MyoD-Cre, Tie2-Cre and smooth muscle myosin heavy chain-Cre (SMMHC-Cre) to trace the possible involvement of skeletal muscle stem cells, endothelial precursors and vascular smooth muscle cells, respectively, in heterotopic muscle ossification [83]. Speer *et al*. [84] used SM22-Cre to genetically trace cells derived from smooth muscle and found that smooth muscle cells gave rise to osteochondrogenic precursor- and chondrocyte-like cells in calcified blood vessels of matrix Gla protein deficient (MGP^−/−^) mice. Recently, tenomodulin, scleraxis and thrombospondin 4 have been suggested to be more specific biomarkers for tendon fibroblasts and were discussed in a review article [85]. Whether these tendon-related markers could be used for tendon lineage tracing and hence for understanding the *in vivo* identity and roles of tendon stem cells needs further experiments. Table 1 summarizes the possible strategies, their advantages and limitations, for tendon stem cell tracking *in vivo*.

**Table 1:** Possible strategies, their advantages and limitation in tendon stem cell tracking *in vivo*

Methods	Advantages	Limitations	References
*In situ* labelling with MSC or tendon stem cell-specific markers	Sensitive	Lack of specific MSC or tendon stem cell makers	[39, 40]
	Simple		
	More than one markers can be used to increase the specificity		
Injection of labelled stem cells into circulation	Results may be affected by injection routes	Non-specific engraftment in other organs such as lung and liver	79
	Universal for the study the distribution and functions of stem cells in different tissues	Tendon has poor blood supply and may affect the homing of stem cells to tendon under healthy condition	
		Specific signals are needed for homing of injected stem cells to tendon	
		Less accurate in studying the natural distribution of tendon stem cells	
		May not reflect the endogenous tendon stem cell activities	
Injection of labelled stem cells into tendon	Specific tissue engraftment	Successful injection in healthy tendon remains unknown	79
	Simple	May not reflect or may even disturb the endogenous stem cell activities	
	Universal for the study the distribution and functions of stem cells in different tissues	Injury may be induced during injection	
BrdU labelling	Universal for the study the distribution and functions of stem cells in different tissues	Non-specific and may have false-positive results	[45]
		Need stem cell markers for further verification	
Detection or tracing telomerase-positive cells	Universal for the study the distribution and functions of stem cells in different tissues	Non-specific	[82]
		Need stem cell markers for further verification	
Genetic-based lineage tracing	Taking information related to tissue development into account in the design of vector for tracing cells derived from different tissues	Lack of specific tendon makers	[32, 83, 84]
	Specific		

## Conclusion

In conclusion, current evidence suggests that tendon stem cells are heterogeneous and could be identified in both peritenon and tendon proper. There were likely both vascular and non-vascular sources of stem cells in tendons. Based on the current *in vitro* data, the fate of tendon stem cells is likely to be regulated by oxygen tension, mechanical loading, composition and topographical cues of extracellular matrix, biological factors such as BMPs and Wnts as well as tenocytes. The exact *in vivo* role of tendon stem cells is unknown, but they might contribute to tendon homeostasis and tendon pathologies *via* both direct cell differentiation and production of trophic factors. Potential strategies for understanding the *in vivo* niche of tendon stem cells have been discussed. Information about the *in vivo* identity of tendon stem cells, if known, would shed light on the design of new drugs for therapeutic manipulation of endogenous tendon stem cells for the re-establishment of a functional niche or for the *ex vivo* amplification and differentiation of tendon stem cells for tendon repair.
